# Identification of Drug Resistance Determinants in a Clinical Isolate of Pseudomonas aeruginosa by High-Density Transposon Mutagenesis

**DOI:** 10.1128/AAC.01771-19

**Published:** 2020-02-21

**Authors:** Michael S. Sonnabend, Kristina Klein, Sina Beier, Angel Angelov, Robert Kluj, Christoph Mayer, Caspar Groß, Kathrin Hofmeister, Antonia Beuttner, Matthias Willmann, Silke Peter, Philipp Oberhettinger, Annika Schmidt, Ingo B. Autenrieth, Monika Schütz, Erwin Bohn

**Affiliations:** a Interfakultäres Institut für Mikrobiologie und Infektionsmedizin Tübingen (IMIT), Institut für Medizinische Mikrobiologie und Hygiene, Universitätsklinikum Tübingen, Tübingen, Germany; b NGS Competence Center Tübingen (NCCT), Institut für Medizinische Mikrobiologie und Hygiene, Universitätsklinikum Tübingen, Tübingen, Germany; c Center for Bioinformatics (ZBIT), Universität Tübingen, Tübingen, Germany; d Interfakultäres Institut für Mikrobiologie und Infektionsmedizin Tübingen (IMIT), Department of Biology, Microbiology & Biotechnology, Universität Tübingen, Tübingen, Germany; e Institut für Medizinische Genetik und Angewandte Genomik, Universitätsklinikum Tübingen, Tübingen, Germany

**Keywords:** *Pseudomonas aeruginosa*, multidrug resistance, antibiotics, TraDIS, clinical isolate, peptidoglycan recycling, AmpC β-lactamase, peptidoglycan

## Abstract

With the aim to identify potential new targets to restore antimicrobial susceptibility of multidrug-resistant (MDR) Pseudomonas aeruginosa isolates, we generated a high-density transposon (Tn) insertion mutant library in an MDR P. aeruginosa bloodstream isolate (isolate ID40). The depletion of Tn insertion mutants upon exposure to cefepime or meropenem was measured in order to determine the common resistome for these clinically important antipseudomonal β-lactam antibiotics. The approach was validated by clean deletions of genes involved in peptidoglycan synthesis/recycling, such as the genes for the lytic transglycosylase MltG, the murein (Mur) endopeptidase MepM1, the MurNAc/GlcNAc kinase AmgK, and the uncharacterized protein YgfB, all of which were identified in our screen as playing a decisive role in survival after treatment with cefepime or meropenem. We found that the antibiotic resistance of P. aeruginosa can be overcome by targeting usually nonessential genes that turn essential in the presence of therapeutic concentrations of antibiotics. For all validated genes, we demonstrated that their deletion leads to the reduction of *ampC* expression, resulting in a significant decrease in β-lactamase activity, and consequently, these mutants partly or completely lost resistance against cephalosporins, carbapenems, and acylaminopenicillins. In summary, the determined resistome may comprise promising targets for the development of drugs that may be used to restore sensitivity to existing antibiotics, specifically in MDR strains of P. aeruginosa.

## Abstract (precis)

With the aim to identify potential new targets to restore antimicrobial susceptibility of multidrug-resistant (MDR) Pseudomonas aeruginosa isolates, we generated a high-density transposon (Tn) insertion mutant library in an MDR P. aeruginosa bloodstream isolate (isolate ID40). The depletion of Tn insertion mutants upon exposure to cefepime or meropenem was measured in order to determine the common resistome for these clinically important antipseudomonal β-lactam antibiotics.

## INTRODUCTION

Pseudomonas aeruginosa is one of the most important pathogens involved in nosocomial infections, such as pneumonia, urinary tract infections, wound infections, and potentially life-threating bloodstream infections. In particular, intensive care and immunocompromised patients are at risk for the development of severe infections. Multidrug-resistant (MDR) strains are emerging, which makes the treatment of P. aeruginosa infections even more difficult. For this reason, WHO ranked carbapenem-resistant P. aeruginosa in the top class of its list of priority pathogens for which new antibiotics are urgently needed (1). For an increasing number of cases, colistin is the last treatment option, despite its neuro- and nephrotoxic side effects.

P. aeruginosa employs various intrinsic and acquired antibiotic resistance mechanisms. The high intrinsic resistance is mainly caused by the very low permeability of the outer membrane (2) and the inducible expression of efflux pumps and enzymes mediating resistance, like AmpC (3). *ampC* is expressed at a low level in wild-type (WT) strains, but its expression can be strongly increased in strains in which *ampC* is derepressed. Derepression of *ampC* is often caused by mutations in the transcriptional regulator AmpR in AmpD (4, 5) or in the *dacB* gene, encoding muropeptide amidase and penicillin-binding protein 4 (PBP4), respectively (6), leading to an increased pool of 1,6-anhydromuropeptides originating from the peptidoglycan (PG) recycling pathway (7). Moreover, *ampC* expression can be induced by β-lactam antibiotics and β-lactamase inhibitors, leading to resistance against most β-lactam antibiotics (8).

One strategy that may be used to reconsider the use of antibiotics that have become ineffective because of the development of resistance is inactivation of the primary resistance mechanism. Thus, the combination of β-lactam antibiotics and β-lactamase inhibitors, such as tazobactam, which block the activity of β-lactamases, makes it possible to reconsider the use of antibiotics such as piperacillin. However, such combinations often fail again to kill microbial pathogens because of β-lactamases which are resistant to the β-lactamase inhibitors (9–11). One upcoming strategy is to use a different class of antibiotic adjuvants. Such adjuvants would not inactivate a primary resistance mechanism but, rather, would act on a secondary resistance gene. Several examples for such a strategy have been described (12–16). In this study, we wanted to find out which proteins can serve as targets to resensitize MDR P. aeruginosa strains to treatment with β-lactam antibiotics.

To answer this question, we performed transposon (Tn)-directed insertion sequencing (TraDIS) using the clinical bloodstream isolate ID40, which is resistant to many β-lactam antibiotics, to assess the resistome of P. aeruginosa by an approach similar to that described by Jana et al. (17). TraDIS has been shown to be a valuable tool under particular conditions and in various approaches to find genes responsible for growth (18–21). We constructed a Tn mutant library in the MDR ID40 strain and subjected it to cefepime (FEP) or meropenem (MEM) treatment. TraDIS revealed nonessential candidate genes, including well-known genes as well as so far unknown genes, whose inactivation breaks the resistance against these antibiotics. Some candidates were verified by testing the respective deletion mutants for their antibiotic sensitivity, β-lactamase activity, and *ampC* expression. The presence of these genes seems to be crucial to achieve or maintain antibiotic resistance. These genes may comprise the most promising nonessential target genes for the development of novel antibiotic adjuvants to reconsider the use of β-lactam antibiotics for the treatment of infections caused by resistant strains of P. aeruginosa.

## RESULTS

### ID40 sequence and resistance profile.

To determine the resistome of an MDR P. aeruginosa strain against β-lactam antibiotics, we used the bloodstream isolate ID40 (22). ID40 belongs to sequence type 252 (ST-252; determined by use of multilocus sequence typing [MLST 2.0, Center for Genomic Epidemiology, DTU, Denmark] [23]) and is resistant to piperacillin (PIP), piperacillin-tazobactam (TZP), cefepime (FEP), ceftazidime (CAZ), aztreonam (ATM), levofloxacin (LEV), ciprofloxacin (CIP), and imipenem (IMP). Moreover, ID40 is intermediate for meropenem (MEM) and sensitive to amikacin (AMI), gentamicin (GEN), tobramycin (TOB), and colistin (COL) (see Table S1 in the supplemental material). The whole genome and the plasmid sequence were annotated and submitted to the European Nucleotide Archive (ENA; https://www.ebi.ac.uk/ena; accession number PRJEB32702).

The ID40 chromosome is 6.86 Mbp in size, encodes 6,409 open reading frames, and carries a plasmid of 57,446 bp comprising 59 putative genes. Resistance genes were searched for using the ResFinder tool (24), revealing the following resistance genes: *aph(3′)-IIb* (*neo*) for aminoglycoside resistance, *bla*_OXA-486_ (*bla*) and *oxa*_PAO1_ (*ampC*, PDC-3) for β-lactam resistance, *crpP* for fluoroquinolone resistance, and *fosA* (*fosA_1*) for fosfomycin resistance. Additionally, we found a point mutation in the *dacB* gene (PBP4; G to A at nucleotide 1310, G437D), which is known to be responsible for resistance against β-lactam antibiotics, as shown by an increased MIC for CAZ from 1 μg/ml to 32 μg/ml in P. aeruginosa PAO1 (6). Therefore, the mutation in *dacB* most likely rationalizes the different resistance level of ID40 in comparison to that of strain PA14, which contains the same resistance genes but is sensitive to all β-lactam antibiotics. Other resistance mechanisms, like the reduced expression of *oprD* and overexpression of efflux pumps, were not specifically addressed, but the possibility of their contribution to resistance cannot be finally excluded. Analysis of the OprD sequence and comparison to the literature did not provide any clear evidence that OprD of ID40 is dysfunctional (25–28).

### Construction of a high-density mutant library and TraDIS sequencing.

Growth of the Tn library in lysogeny broth (LB) revealed approximately 100,000 unique Tn insertions distributed across the genome, with an average of 18 Tn insertion sites per 1 kbp of coding sequence. The homogeneous distribution of the Tn insertions and the homogeneous coverage of the whole genome are shown in Fig. S1.

Analysis of the unchallenged Tn library showed that of 6,468 genes, 697 genes were determined to be essential for viability (10.8%) (Data Set S1) and 9 were determined to be ambiguous (0.14%) (Data Set S2). Among these, many genes, for example, *dnaA*, *gyrB*, or *lolA*, were previously described to be essential (29, 30).

### Identification of genes important for resistance against meropenem and cefepime.

**FIG 1:**
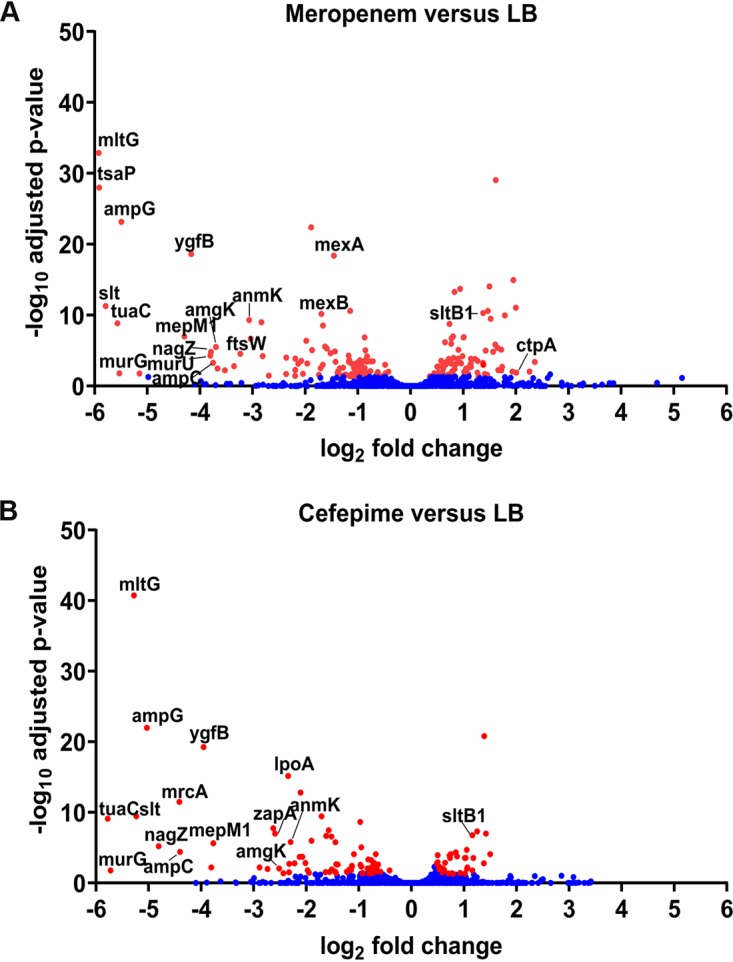
Resistome of the MDR strain ID40 determined by TraDIS. The bacteria comprising the ID40 Tn library were grown in LB with or without 2 μg/ml MEM (A) or 8 μg/ml FEP (B) in 3 independent experiments, and then the DNA of the surviving bacteria was used for sequencing of the Tn-genome junctions. The fold change in expression and adjusted *P* value for samples grown in antibiotics in comparison to those for samples grown in LB were calculated with DESeq2 software for all annotated genes. All genes with read counts significantly different (adjusted *P* value, <0.05) from those for the LB control are colored in red.

The contribution of nonessential genes to antimicrobial resistance was measured by quantifying the depletion of Tn insertion mutants upon exposure to FEP and MEM at the respective breakpoint concentration, defining a P. aeruginosa strain to be sensitive according to EUCAST breakpoints (for FEP, 8 μg/ml; for MEM, 2 μg/ml). For analysis of the TraDIS results, we chose only genes in which the read number in the LB control was >10 in all three independent experiments and which additionally showed a significant change in read counts upon treatment with an adjusted *P* value of <0.05 (Data Set S3). Genes that showed a significant change in read counts in comparison to those for the untreated sample are visualized in Fig. 1. In total, 140 genes fulfilled these criteria upon MEM treatment and 102 genes fulfilled these criteria upon FEP treatment.

Nonessential genes in which the read counts for Tn insertion were reduced at least 5-fold with a high level of significance (adjusted *P* value < 0.05) are listed in Table 1. In total, 24 such genes were identified. Thirteen of those genes fulfilled these criteria for both MEM and FEP, 5 fulfilled these criteria only for MEM, and 6 fulfilled these criteria only for FEP. Most genes were found to be involved in PG synthesis and recycling. The most interesting genes identified in this screening were those which showed a significant reduction in read counts after both MEM and FEP treatment. All TraDIS sequence data were uploaded to ENA (accession number PRJEB32702).

**TABLE 1:** Meropenem and cefepime resistome in P. aeruginosa ID40^*a*^

Category	Identifier	Gene	Name/function	MEM vs LB		FEP vs LB		Orthologue(s)
				Ratio	*P* value	Ratio	*P* value	
Genes with an adjusted *P* value of <0.05 and a ≥5-fold reduction for MEM and FEP								
Resistance	TUEID40_04486	*ampC*	β-Lactamase	0.07	0.00052	0.05	3.87E−5	PA14_10790, PA4110
PG synthesis/recycling	TUEID40_05675	*slt*	Soluble lytic transglycosylase	0.02	5.08E−12	0.03	3.61E−10	PA14_25000, PA3020
	TUEID40_05736	*mltG*	Endolytic murein transglycosylase	0.02	1.32E−33	0.03	1.77E−41	PA14_25730, PA2963
	TUEID40_04290	*mepM1*	Murein dd-endopeptidase	0.05	1.01E−07	0.07	2.29E−06	PA14_08540, PA0667
	TUEID40_02325	*ftsW*	Synthesis of septal peptidoglycan during cell division	0.11	2.76E−05	0.20	0.0019	PA14_57360, PA4413
	TUEID40_02305	*ampG*	Permease	0.02	7.00E−24	0.03	1.01E−22	PA14_57100, PA4393
	TUEID40_05690	*nagZ*	β-*N*-Acetyl-d-glucosaminidase	0.07	1.56E−05	0.04	6.23E−06	PA14_25195, PA3005
	TUEID40_04289	*anmK*	Anhydro-*N*-acetylmuramic acid kinase	0.12	4.938E−10	0.20	1.62E−06	PA14_08520, PA0666
	TUEID40_04233	*amgK*	*N*-Acetylmuramate/*N*-acetylglucosamine kinase	0.08	3.05E−06	0.17	0.0085	PA14_07780, PA0596
	TUEID40_04234	*hddC* or *murU*	Similar to *N*-acetylmuramate alpha-1-phosphate uridylyltransferase MurU of Pseudomonas putida	0.07	5.10E−05	0.15	0.0001	PA14_07790, PA0597
LPS	TUEID40_05537	*wbpE*	UDP-2-acetamido-2-deoxy-3-oxo-d-glucuronate aminotransferase	0.10	1.58E−03	0.135	6.43E−03	PA3155
Unknown	TUEID40_03245	*ygfB*	YgfB-like proteins, unknown	0.06	2.35E−19	0.06	5.56E^−^20	PA14_69010, PA5225
	TUEID40_05543	*tuaC*	Glycosyltransferase family 1	0.02	1.35E−09	0.02	7.79E−10	
Genes with an adjusted *P* value of <0.05 and a ≥5-fold reduction only for MEM								
PG synthesis/recycling	TUEID40_04881	*mepM2*	Murein dd-endopeptidase MepM, unknown function	0.14	3.60E−12	0.37	0.002	PA14_15100, PA3787
Type IV pili assembly	TUEID40_03621	*tsaP*	Type IV pilus secretin-associated protein; anchors the outer membrane type IV pili secretin complex to the peptidoglycan	0.02	1.02E−28	0.31	3.61E−10	PA14_00210, PA0020
β-Barrel assembly	TUEID40_01638	*bepA* or *ygfC_1*	β-Barrel assembly enhancing protease	0.12	2.17E−07	0.24	1.20E−06	PA14_51320, PA1005
Unknown	TUEID40_03216		Putative zinc protease	0.14	6.06E−05	0.21	0.001	PA14_68640, PA5196
	TUEID40_05674		Uncharacterized conserved protein YecT, DUF1311 family	0.19	9.84E−05	0.74	1.0	PA14_24990, PA3021
Genes with an adjusted *P* value of <0.05 and a ≥5-fold reduction only for FEP								
PG synthesis/recycling	TUEID40_05519	*gph_2* or *mupP*	*N*-Acetylmuramic acid 6-phosphate phosphatase MupP	0.27	0.14	0.185	4.12E−02	PA14_23210, PA3172
	TUEID40_03006	*mrcA*	Penicillin-binding protein 1	0.73	0.40	0.05	3.15E−12	PA14_66670, PA5045
	TUEID40_02335	*lpoA*	Penicillin-binding protein activator	1.03	1	0.20	6.87E−16	PA14_57480, PA4423
Cell division	TUEID40_03247	*zapA*	Cell division protein ZapA	0.39	0.00017	0.17	1.00E−07	PA14_69030, PA5227
Porin	TUEID40_00776	*oprF*	Outer membrane protein F	0,22	0.036	0.20	0.03	PA14_41570, PA1777
Unknown	TUEID40_01298		Uncharacterized putative membrane-bound pyrroloquinoline quinone-dependent dehydrogenase	0.45	0.0018	0.16	1.80E−08	PA14_47350, PA1305

a Data are for genes for which insertion sequence abundance was significantly reduced (>5-fold; adjusted *P* value, <0.05) upon exposure to 2 μg/ml MEM or 8 μg/ml FEP. Differences in insertion sequence abundance are expressed as the mean of the ratio of the normalized sequence read numbers of antibiotic-treated culture in relation to the normalized sequence read numbers of the LB control culture of the Tn library. In total, three independent experiments were performed.

We found several genes dedicated to PG recycling metabolism, such as *ampG* and *nagZ*, known to be important for resistance against β-lactam antibiotics (31–36). In addition, the efflux pump genes *mexA* and *mexB* (Data Set S3) as well as the porin OprF were also identified in our screen and have been described to be involved in antibiotic resistance (37) (Table 1). This finding points out that our approach can identify nonessential genes involved in antibiotic resistance.

A pathway that connects cell wall recycling to PG *de novo* biosynthesis is responsible for the intrinsic resistance of P. aeruginosa to fosfomycin, inhibiting the synthesis of PG by blocking the formation of *N*-acetylmuramic acid (MurNAc) (38–41). This cell wall salvage pathway comprises anhydro-MurNAc kinase (AnmK), an anomeric cell wall amino sugar kinase (AmgK), MurNAc-6-phosphatase (MupP), and an uridylyltransferase (MurU), together converting 1,6-anhydro-*N*-acetylmuramic acid (AnhMurNAc) to UDP-MurNAc, thereby bypassing the fosfomycin-sensitive *de novo* synthesis of UDP-MurNAc. We identified the genes for all four of these enzymes (Table 1) and conclude that the anabolic recycling pathway may play a critical role in maintaining resistance against β-lactam antibiotics, at least in strains with high levels of β-lactamase activity.

Moreover, genes encoding the lytic transglycosylases (LTs) Slt and MltG were found to be associated with resistance upon treatment with MEM and FEP (Table 1). The loss of Slt was shown to reduce resistance against β-lactam antibiotics in PAO1 (42). MltG was described as one of several LTs to be inhibited by bulgecin, a sulfonated glycopeptide originally isolated from P. acidophila and P. mesoacidophila, resulting in slightly reduced MICs of CAZ and MEM (16).

MepM1 (YebA, PA0667) belongs to a group of murein endopeptidases (EPs) which putatively modulate PG cross-linking (43). A study revealed that the protease CtpA (PA5134) inactivates various EPs, namely, those encoded by PA0667 (TUEID40_04290 or *mepM1*), PA4404 (TUEID40_02316), PA1198 (TUEID40_01415), and PA1199 (TUEID40_01414), and thereby controls the level of PG cross-linking (43). TUEID40_01415 also showed reduced read counts upon treatment with MEM and/or FEP, but to a much lesser extent than MepM1 did (Data Set S3). In addition, the EP MepM2, which is not regulated by CtpA, at least in the P. aeruginosa PAK strain (43), also seems to be involved in maintaining antibiotic resistance (Table 1).

Furthermore, we identified two so far unknown or uncharacterized candidate genes putatively involved in antibiotic resistance against both MEM and FEP: TUEID40_05543 (*tuaC*) belongs to the glycosyltransferase 1 family, and TUEID40_03245 encodes a YgfB-like protein with a so far unknown function which is referred to here as YgfB.

### Confirmation of selected genes involved in antimicrobial resistance.

To validate our TraDIS results, deletion mutants for *mltG*, *mepM1*, *amgK*, *ygfB*, *tuaC*, and *ctpA*, as well as a *ctpA mepM1* double mutant, were tested for their sensitivity against β-lactam antibiotics. Broth microdilution assays indicated that the deletion of *mltG*, *mepM1*, *ygfB*, and *amgK* reduced the MIC values for all tested β-lactam antibiotics (Table 2) except for IMP (Δ*mepM1* mutant) and MEM (Δ*mepM1* and Δ*amgK* mutants), while deletion of *tuaC* resulted in only a slight reduction in the MIC for TZP. The MIC values were reduced below the breakpoint for FEP and ATM in the Δ*mltG*, Δ*mepM1*, Δ*ygfB*, and Δ*amgK* mutants and for CAZ in the Δ*mltG* and Δ*amgK* mutants. Additionally, the Δ*mltG* mutant showed MICs below the breakpoint for PIP, TZP, and IMP. These data confirm the validity of the TraDIS screen and demonstrate the contribution of these genes to resistance against β-lactam antibiotics in the ID40 strain.

**TABLE 2:** Susceptibility of ID40 WT and deletion mutants to β-lactam antibiotics^*a*^

Antibiotic	MIC (mg/liter)										
	Breakpoint		ID40 WT	Δ*mltG* mutant	Δ*mepM1* mutant	Δ*ctpA* mutant	Δ*mepM1* Δ*ctpA* mutant	Δ*ygfB* mutant	Δ*amgK* mutant	Δ*tuaC* mutant	PA14
	Susceptible	Resistant									
MEM	2	8	8	**4**	8	*16*	*16*	**4**	8	8	<0.125
IMP	4	4	32	**4**	32	32	32	**8**	**8**	32	<1
FEP	8	8	16	**4**	**4**	*32*	*32*	**8**	**8**	16	<1
CAZ	8	8	32	**2**	**16**	32	32	**16**	**8**	32	<1
PIP	16	16	128	<**4**	**64**	*>128*	128	**32**	**32**	128	<4
TZP	16	16	128	**4**	**32**	128	128	**32**	**32**	**64**	4
ATM	16	16	32	**2**	**16**	*>32*	32	**16**	**8**	*>32*	8
FOS			96	96	96	96	**64**	*128*	**48**	96	48

a MICs of ID40 WT and deletion mutant strains were determined by broth microdilution or by Etest for fosfomycin. Susceptible was an MIC less than or equal to the breakpoint, and resistance was an MIC greater than the breakpoint. MIC values for the deletion mutants lower than the MIC for the ID40 WT are in bold, and MICs below the MIC breakpoint are in bold and underlined. MIC values higher than the MIC for the ID40 WT are in italics. MEM, meropenem; IMP, imipenem; FEP, cefepime; CAZ, ceftazidime; PIP, piperacillin; TZP, piperacillin-tazobactam; ATM, aztreonam; FOS, fosfomycin.

Deletion of *ctpA* increased the MIC values for MEM, FEP, PIP, and ATM. Thus, we hypothesize that the increased activity of MepM1 and other CtpA substrates leads to increased resistance. The MIC values of PIP and ATM for the Δ*ctpA* Δ*mepM1* double mutant were lower than those for the Δ*ctpA* mutant but higher than those for the Δ*mepM1* deletion mutant, indicating that the other substrates of CtpA might also contribute to resistance against β-lactam antibiotics and compensate for the loss of MepM1 without the inactivation of CtpA. According to the TraDIS data, the most promising CtpA-regulated substrates which may, in combination with MepM1, contribute to β-lactam resistance are TUEID40_02316 and TUEID40_01415 (Data Set S3). Furthermore, it could be confirmed that the deletion of *amgK* resulted in reduced resistance against fosfomycin (Table 2 and Table S1), as previously described (39).

For complementation, conditional mutants (the Δ*mltG*::*mltG*, Δ*mepM1*::*mepM1*, Δ*ctpA*::*ctpA*, and Δ*ygfB*::*ygfB* mutants) in which the complemented genes were under the control of a rhamnose-inducible promoter were generated. Complementation could be achieved in the presence of 0.1% rhamnose (Table S1).

### MltG, MepM1, AmgK, and YgfB contribute to β-lactam resistance in ID40 by promoting *ampC* expression.

**FIG 2:**
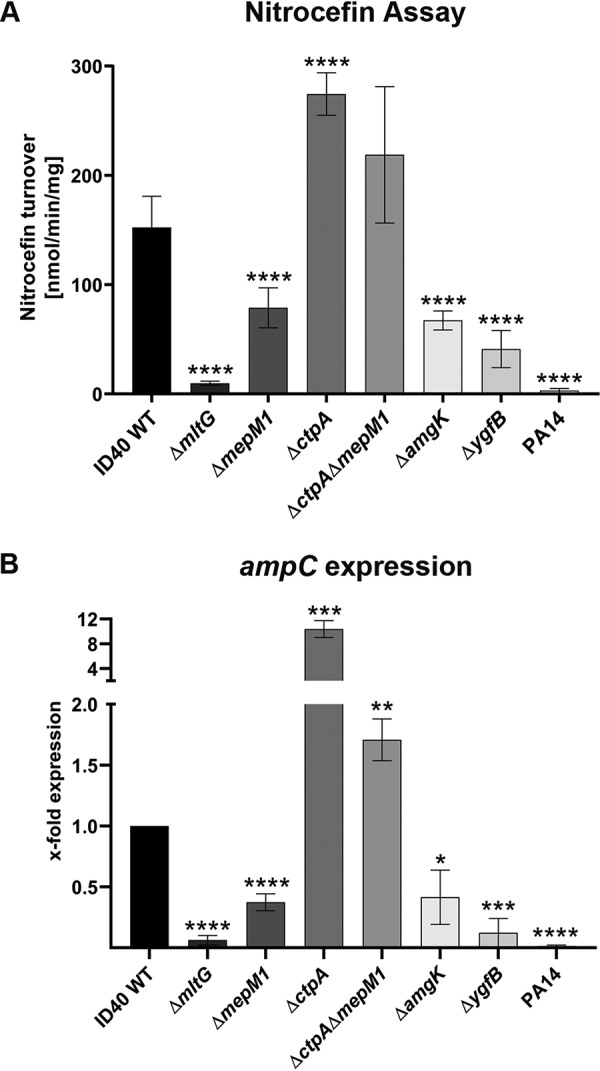
β-Lactamase activity and *ampC* expression in selected deletion mutants. WT and deletion mutant strains were subcultured, and β-lactamase activity was measured by a nitrocefin turnover assay (A) or expression of the *ampC* β-lactamase gene was determined by qRT-PCR (B) in at least 3 independent experiments. Graphs depict the means and SDs. Student's *t* test was performed for determination of the difference in the results for each mutant strain in comparison to those for the WT. *, *P* < 0.05; **, *P* < 0.01; ***, *P* < 0.001; ****, *P* < 0.0001.

To assess in more detail the reason why the mutants show restored susceptibility to β-lactam antibiotics, we measured the β-lactamase activity of ID40, the different deletion mutants, as well as the laboratory strain PA14, which is sensitive to all tested antibiotics (Table 2). As determined by a nitrocefin-based assay, β-lactamase activity was strongly reduced in the Δ*mltG*, Δ*mepM1*, Δ*ygfB*, and Δ*amgK* mutants, with the most profound reduction being seen in the Δ*mltG* mutant, showing β-lactamase activity almost as low as that of the PA14 strain (Fig. 2A) and being sensitive to all β-lactam antibiotics tested (Table S1). The β-lactamase activity corresponded directly to the MIC values for the different mutants. Similarly, higher β-lactamase activity was found in the hyperresistant Δ*ctpA* mutant. Therefore, the changes in MICs for the mutants from those for the ID40 wild type are presumably caused by altered β-lactamase activity. No significant change in β-lactamase activity was found between the Δ*ctpA* Δ*mepM1* mutant and the Δ*ctpA* mutant, indicating that the uncontrolled levels of other CtpA substrates can compensate for the lack of MepM1.

The ID40 genome encodes two β-lactamases (*ampC* and *bla*_OXA-486_ [*bla* or *poxB*]). For PoxB, it has been shown that it does not contribute to β-lactam resistance (44). We quantified the expression level of *ampC* to investigate whether the lower β-lactamase activity is due to reduced *ampC* expression. Semiquantitative reverse transcription-PCR revealed that the deletion of *mltG*, *mepM1*, *amgK*, or *ygfB* significantly decreased *ampC* mRNA expression (Fig. 2B). Deletion of *ctpA*, presumably resulting in a higher level of MepM1 and its other substrates, caused an increase in *ampC* expression. The expression level of *ampC* in the different mutants was in agreement with the levels of β-lactamase activity and the MICs of β-lactam antibiotics that we measured. These results indicate that the different levels of resistance of the ID40 mutants are due to different levels of *ampC* expression.

## DISCUSSION

Here, we report the first application, to our knowledge, of TraDIS to an MDR Pseudomonas aeruginosa strain and the evaluation of its nonessential resistome upon exposure to two clinically relevant β-lactam antibiotics. The identified genes might represent targets that could be exploited to resensitize resistant strains so that infections caused by these strains may be treated with β-lactam antibiotics.

**FIG 3:**
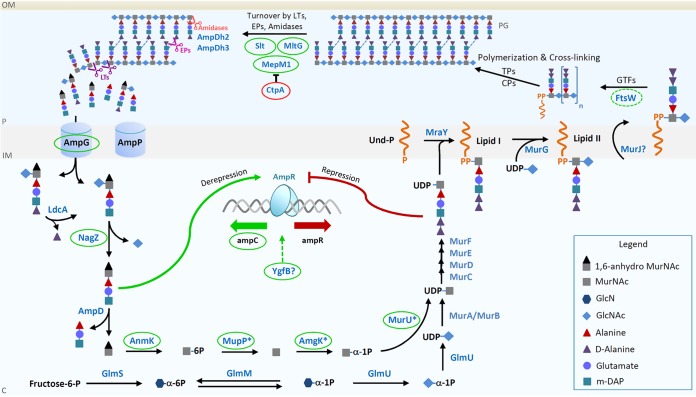
Simplified scheme of PG recycling and synthesis pathway of P. aeruginosa and illustration of the proteins identified by TraDIS. The bacterial murein matrix is formed by chains of the two alternating amino sugars MurNAc (M) and GlcNAc (G), which are linked by β(1→4) glycosidic bonds. Attached to the MurNAc residues is a pentapeptide side chain which typically is composed of l-alanine–γ-d-glutamate–*meso*-diaminopimelic acid–d-alanyl-d-alanine (l-Ala–γ-d-Glu–*m*-DAP–d-Ala-d-Ala). Cross-links between adjacent glycans are mainly built by connecting the *meso*-diaminopimelic acid of one chain with the d-Ala of the other chain. PG synthesis starts in the cytoplasm, where fructose-6-phosphate is converted in several steps by GlmS, GlmM, and GlmU to UDP-GlcNAc. UDP-GlcNAc is further converted to UDP-MurNAc by Mur enzymes A and B, and subsequently, a peptide chain is added by Mur ligases C, D, E, and F to form UDP-MurNAc-pentapeptide. An alternative route to generate UDP-MurNAc-pentapeptide starts with the transfer of GlcNAc-1,6-anhMurNAc-peptides (muropeptides) along with GlcNAc-anhMurNAc into the cytoplasm by the permease AmpG. Some muropeptides (however, not GlcNAc-1,6-anhMurNAc-peptides) or free peptides may also be transported through AmpP, but its function in cell wall recycling has not been elucidated so far. The imported muropeptides are subsequently degraded by NagZ, l,d-carboxypeptidase LdcA, and AmpD, producing d-Ala, GlcNAc, l-Ala–iso-d-glutamate–*meso*-diaminopimelic acid tripeptide, and 1,6-anhMurNAc. AnmK then catalyzes the phosphorylation of 1,6-anhMurNAc, generating MurNAc-6P, which is further processed by MupP and the sugar kinase AmgK to MurNAc-α-1P. The uridylyltransferase MurU then converts the latter to UDP-MurNAc, following the formation of UDP-MurNAc-pentapeptide. The phospho-MurNAc-pentapeptide moiety is then transferred by the cytosolic translocase MraY to the lipid carrier undecaprenol phosphate (Und-P) to generate lipid I, which is subsequently catalyzed by MurG to lipid II by adding GlcNAc to it. Lipid II is then flipped into the periplasm (likely by the putative flippase MurJ), where GlcNAc-MurNAc-peptides are integrated into the growing PG by high-molecular-mass penicillin-binding proteins; glycosyltransferases (GTFs), such as FtsW and RodA; transpeptidases (TPs); and dd-carboxypeptidases (CPs). Low-molecular-mass penicillin-binding proteins, endopeptidases (EPs; such as MepM1), lytic transglycosylases (such as MltG and Slt), and amidases (such as AmpDh2 and AmpDh3) finally cleave the existing PG layer to facilitate the insertion of new glycan strands and simultaneously to release the PG degradation products from the matrix into the cytoplasm. Under normal conditions, the PG precursor UDP-MurNAc-pentapeptide binds to AmpR, causing the repression of *ampC* transcription. In the case of β-lactam treatment, the turnover of the muropeptides is increased (by blockage of PG cross-links), resulting in the accumulation of 1,6-anhMurNAc-pentapeptide in the cytoplasm. The 1,6-anhMurNAc-muropeptides are able to displace UDP-MurNAc-pentapeptides from AmpR, causing the derepression and, hence, activation of *ampC* transcription. YgfB also modulates *ampC* expression, contributing finally to β-lactam resistance, but its specific role in mediating antibiotic resistance remains to be investigated. The proteins found via TraDIS are highlighted with a circle in red for proteins mediating repression of *ampC* expression and with a circle in green for proteins mediating derepression of *ampC* expression. The putative FtsW protein (so far not verified in P. aeruginosa) and the unknown mechanism of YgfB are labeled with interrupted lines. OM, outer membrane; P, periplasm; IM, inner membrane; C, cytoplasm; PG, peptidoglycan; CPs, dd-carboxypeptidases; GTFs, glycosyltransferases; EPs, endopeptidases; LTs, lytic transglycosylases; *, AmgK, MupP, and MurU cell wall recycling enzymes found in P. aeruginosa but not in enterobacteria, such as E. coli (38, 40, 41).

Many of the genes important for high-level β-lactam resistance found in the TraDIS approach are part of the PG recycling pathway of P. aeruginosa (45), showing its critical role for β-lactam resistance in ID40 (46). A simplified scheme of the PG recycling and synthesis pathway of P. aeruginosa and the genes identified by the TraDIS approach as well as genes described to modulate resistance against β-lactam antibiotics is summarized in Fig. 3.

### Players in the periplasm.

The precursors of the PG catabolites contributing to the transcriptional regulation of *ampC* are generated in the periplasm. LTs (such as MltG and Slt), together with low-molecular-mass penicillin-binding proteins, EPs (such as MepM1), and amidases (such as AmpDh2 and 3), cleave the PG layer to facilitate the insertion of new glycan strands and simultaneously release PG degradation products from the matrix into the cytoplasm (45).

Upon treatment with antibiotics, the strongest impact on LTs in the screening was found for *mltG* and *slt*. In addition, and in agreement with the findings of previous studies (16, 42, 47), we also found that the LTs *mltF* and *mltD* maintain resistance, but to a lesser extent than *slt* and *mltG* (see Data Set S3 in the supplemental material). On the other hand, *sltB* and *sltH* seem to counteract resistance (Data Set S3). The recently described MltG may act as a terminase and determine PG chain length (48). Deletion of *mltG* in ID40 significantly reduced *ampC* expression and, consequently, β-lactamase activity and broke resistance against IMP, FEP, CAZ, PIP, TZP, and ATM. These findings confirm the validity of our study and underline the importance of MltG for the induction of *ampC* expression in ID40. As previously demonstrated, MltG, Slt, and MltD are targets of the LT inhibitor bulgecin, reducing the MIC against β-lactam antibiotics (16). According to our data, LTs represent one of the most promising targets for resensitization to treatment with β-lactam antibiotics.

EPs may also contribute to the induction of *ampC* expression. As demonstrated previously, the protease CtpA inactivates and thereby determines the levels of four EPs that control PG cross-linking (43). Of this group, *mepM1* showed the highest reduction of Tn insertion read counts when treatment with antibiotics and the control were compared, while Tn insertions in PA1198 (TUEID40_01415) had a minor impact on growth in the presence of MEM. In addition, *mepM2* (TUEID40_04881), a further EP which is not regulated by CtpA in strain PAK (43), also seems to contribute to resistance against β-lactam antibiotics. While deletion of *mepM1* leads to reduced MIC values of β-lactam antibiotics, deletion of *ctpA* leads to hyperresistance, probably by deregulating the levels of its substrates. The role of deleted or nonfunctional CtpA in mediating hyperresistance is further supported by the findings of a study by Sanz-García et al., who showed that upon ceftazidime-avibactam treatment, mutations in the *ctpA* gene emerge and that these lead to resistance (49). Additional deletion of *mepM1* in the *ctpA* mutant reduces MIC values compared to those for the Δ*ctpA* mutant for PIP and ATM but results in still higher MIC values compared to those for the *mepM1* deletion mutant, indicating that other CtpA-dependent EPs also contribute to the upregulation of *ampC* expression. These data suggest that the high level of activity of MepM1 promotes increased *ampC* expression. Thus, inhibition of several of these EPs could be a possible way to break antibiotic resistance.

### Players in the cytoplasm.

After PG catabolites have been formed in the periplasm, they are transported into the cytoplasm by the permease AmpG and partly by AmpP (50). In the following process, the 1,6-anh-MurNAc-peptides are degraded by LdcA, NagZ, and AmpD. The amidase AmpD cleaves the peptide chain attached to 1,6-anhMurNAc so that the generated 1,6-anhMurNAc can subsequently be recycled to UDP-MurNAc by the so-called cell wall salvage pathway via AnmK, MupP, AmgK, and MurU, which bypasses the *de novo* biosynthesis of UDP-MurNAc (38, 39). Finally, UDP-MurNAc is modified by the Mur enzymes to form UDP-MurNAc-pentapeptide (45). Both 1,6-anhMurNAc-peptides and UDP-MurNAc-pentapeptide can bind to the *ampC* regulator AmpR. Thereby, 1,6-anhMurNAc-peptides induce *ampC* expression, while UDP-MurNAc-pentapeptide bound to AmpR represses *ampC* expression.

As observed in our TraDIS data and as also shown previously, the loss of AmpG or NagZ results in decreased amounts of 1,6-anhMurNAc-peptides and hence results in increased susceptibility to β-lactam antibiotics (32, 47). On the other hand, the loss of AmpD leads to the accumulation of 1,6-anhMurNAc-peptides and, therefore, increased *ampC* expression (51) and is a frequent cause of high levels of *ampC* expression in clinical isolates of P. aeruginosa (52, 53).

### Players of the cell wall salvage pathway.

The individual deletion of each of the 4 genes (*anmK*, *mupP*, *amgK*, and *murU*) of the cell wall salvage pathway in PAO1 has been shown to lead to increased β-lactamase activity and a subtle increase in the levels of resistance against cefotaxime and CAZ (41). Although this effect cannot be explained so far, it was proposed that it might be due to the reduction of the steady-state level of the *ampC* repressor UDP-MurNAc-pentapeptide. Consequently, 1,6-anhMurNAc-peptides would be more likely to bind to AmpR and thereby induce *ampC* expression (41). In contrast, another study showed that the deletion of *amgK* also in P. aeruginosa PAO1 had no impact on CAZ or IMP resistance (39), which could be confirmed in our study for all β-lactam antibiotics tested (Table S1). Interestingly, in our study we observed that Tn insertions in all genes of the MurU pathway reduced the level of β-lactam resistance. Validation of the screening results using an *amgK* deletion mutant confirmed these results. This finding is indeed counterintuitive, and more detailed explorations are necessary to clarify this issue. Presumably, the anabolic recycling pathway somehow counteracts the derepression of *ampC* in the *dacB* background of ID40.

### Uncharacterized players.

Additionally, we identified several uncharacterized genes in the TraDIS screening whose results are presented here. Since deletion of the gene *tuaC* showed only a slight reduction in the MICs against some β-lactam antibiotics, we focused on TUEID40_03245, which we termed *ygfB* due to its similarity to the homologous gene in Escherichia coli. Deletion of *ygfB* resulted in decreased *ampC* expression and β-lactamase activity and broke resistance against FEP and ATM in ID40. To our best knowledge, this gene has so far not been described in the context of antibiotic resistance. *ygfB* is located in an operon together with the *pepP*, *ubiH*, a PA14_68970 orthologue, and *ubiI. ubiI* and *ubiH* are essential genes important for ubiquinone biosynthesis. Similar operon structures are found also in E. coli, Acinetobacter baumannii, and Legionella pneumophila. P. aeruginosa YgfB shares 33% identical amino acids with E. coli and A. baumannii YgfB and 32% with L. pneumophila YgfB. Interestingly, the aminopeptidase gene *pepP*, which exists in a region adjacent to *ygfB*, was also identified in the TraDIS screening, but Tn insertion read counts indicate that a lack of *pepP* might contribute to hyperresistance.

Moreover, experiments with PAO1 Tn mutants suggested that P. aeruginosa YgfB may contribute to virulence in a Caenorhabditis elegans infection model (54). In addition, a TraDIS experiment suggested that the *ygfB* orthologue PA14_69010 may play a role in effective colonization in the cecum of mice (55). Thus, the possible role in virulence as well as the ability to modulate antibiotic resistance could mean that this gene is of interest as a target for the development of antibiotic adjuvants which might additionally reduce virulence. In further studies, we will address the function of YgfB and its specific role in mediating antibiotic resistance.

In conclusion, using TraDIS we identified a set of nonessential genes which are crucial for the induction of *ampC* expression and β-lactam resistance. As shown in a recent study, overexpression of *ampC* is the most frequent cause for the development of resistance in strains capable of expressing *ampC*, as shown by the acquisition of mutations in *dacB*, *ampD*, and *mpl* after exposure of the P. aeruginosa PAO1 WT to increasing concentrations of ceftazidime (56). However, there are additional mechanisms for the development of resistance against β-lactam antibiotics which gain more importance when *ampC* expression is hindered. Mutations in *ftsI* leading to a modification of PBP3 (the target of β-lactam antibiotics), mutations, or overexpression of the efflux pump MexAB-OprM, as well as large chromosomal deletions, led to resistance against ceftazidime, albeit to a lower level than β-lactamase-dependent resistance (56). This aspect will have to be considered for the development of adjuvants leading to the decreased expression of *ampC*.

Nevertheless, the genes identified in our study provide promising candidates as targets for the development of novel adjuvants to restore the function of β-lactam antibiotics in MDR P. aeruginosa strains with high levels of AmpC activity.

## MATERIALS AND METHODS

### Bacterial strains and culture conditions.

The bacterial strains and plasmids used in this study are listed in Table S2 in the supplemental material. Bacteria were cultivated overnight at 37°C with shaking at 200 rpm in lysogeny broth (LB) containing suitable antibiotics, if necessary. Overnight cultures were diluted 1:20 into LB containing suitable antibiotics or additives, like l-rhamnose, and grown for 3 h at 37°C and 200 rpm. The growth of bacteria in LB at 37°C in a 24-well plate was measured using a Tecan Infinite 200 Pro plate reader.

### Whole-genome sequencing of the ID40 isolate.

DNA isolation, library preparation, and Illumina sequencing of the ID40 strain have been described by Willmann et al. (22).

For Nanopore sequencing, the DNA was isolated using a DNeasy UltraClean microbial kit (Qiagen). Library preparation was conducted using a ligation sequencing kit (Oxford Nanopore Technologies). Sequencing was performed on a PromethION sequencer (Oxford Nanopore Technologies) on a FLO-PRO002 flow cell (version R9).

The ID40 genome was assembled using a hybrid assembly approach that combines the Nanopore long reads with exact Illumina short reads. We used the hybrid assembly pipeline pathoLogic (57) with default settings and selected Unicycler (58) as the main assembly algorithm. Further manual scaffolding yielded a single circular plasmid and a circular chromosome. The assembled genome as well as the plasmid sequence was annotated using the Prokka (version 1.11) software tool (59, 60).

### Generation of ID40 Tn library.

The ID40 Tn mutant library was generated as described previously (55, 61) with some modifications. The donor strain E. coli SM10 λ *pir* containing pBT20 was grown in LB containing 15 μg/ml gentamicin (Gm), and recipient strain ID40 was grown in LB. Cell suspensions of both strains were adjusted to an optical density at 600 nm (OD_600_) of 2.0 and mixed, and droplets of 100 μl were spotted onto prewarmed LB agar plates. After incubation at 37°C for 3 h, the mating mixtures were scraped off the plate and resuspended in 12 ml LB. Aliquots of 100 μl were plated onto 100 LB agar plates containing 25 μg/ml irgasan and 75 μg/ml Gm. After overnight growth at 37°C, all colonies (approximately 5,000 per plate) were scraped off the LB agar, resuspended, and washed once in LB. To eliminate satellite colonies, 1 liter of LB containing 75 μg/ml Gm was inoculated with the suspension to an OD_600_ of 0.1 and grown to an OD_600_ of 1.2. The bacteria were washed once and adjusted to an OD_600_ of 22 in LB containing 20% glycerol, and finally, aliquots of 1 ml were frozen at −80°C.

### Tn library antibiotic exposure.

One aliquot of the Tn library was centrifuged, resuspended in LB, and grown in 100 ml LB overnight. The overnight cultures were diluted 1:100 into 100 ml LB with or without 8 μg/ml FEP or 2 μg/ml MEM and grown at 37°C. After 24 h, the cultures were diluted 1:100 into fresh LB and grown for another 24 h at 37°C to enrich the viable bacteria.

### Library preparation for TraDIS.

Genomic DNA of 5 × 10^9^ bacteria per sample was isolated using a DNeasy UltraClean microbial kit (Qiagen).

Two micrograms of DNA per sample was sheared into fragments of 300 bp with an M220 Focused-Ultrasonicator (Covaris), and a cleanup was conducted with a 1.5-fold volume of Agencourt AMPure XP beads (Beckman Coulter). End repair, A tailing, and adapter ligation were done using an NEBNext Ultra II DNA library preparation kit for Illumina (NEB). A splinkerette and the P7 indexed primer were used as adapters, leading to the enrichment of Tn-containing fragments in the PCR (62–64). Fragments were size selected using Agencourt AMPure beads and amplified by PCR with one Tn-specific primer and one index primer (Illumina) in 20 cycles using Kapa HiFi HotStart ReadyMix (Kapa Biosystems). The proper size distribution and quality of the samples were assessed with an Agilent DNA high-sensitivity kit on a 2100 bioanalyzer (Agilent Technologies). After a final cleanup, the concentration of total fragments and of Tn-containing fragments was measured by quantitative PCR (qPCR) using a Kapa SYBR Fast qPCR master mix (2×) kit (Kapa Biosystems) with one P5-specific primer and one P7-specific primer or one Tn-specific primer and one P7-specific primer, respectively.

### Sequencing.

Samples were adjusted to 4 nM in resuspension buffer (Illumina), pooled, and denatured with 0.2 N NaOH. Subsequently, the library was diluted to 8 pM in hybridization buffer (Illumina) and sequenced with a MiSeq reagent kit (version 2; 50 cycles) on a MiSeq sequencer (Illumina) with a bacteriophage phiX (Illumina) spike in of 5% and dark cycles (62).

### TraDIS data analysis.

Sequencing reads containing the Tn tag were mapped against the ID40 reference genome, using the Bio::TraDIS pipeline (62), in order to determine the locations and numbers of Tn insertions. For each gene, an insertion index was calculated by dividing the number of insertions in a gene by the total gene length. The bimodal distribution of insertion indices allows the determination between essential and nonessential genes, as recently described (15, 65). Genes that fulfilled the cutoff criterion of an insertion index of <0.0019 for essential genes or an insertion index of >0.0026 for nonessential genes were categorized in these groups. All other genes were considered ambiguous (Data Set S2).

Statistical analysis was performed using DESeq2 software (https://bioconductor.org) (66). Differential gene expression analysis was performed for group comparisons of MEM versus the control and FEP versus the control. Genes were categorized as differentially enriched or depleted if the adjusted *P* value was <0.05.

### Generation of in-frame deletion mutants.

In-frame deletion mutants were generated using the suicide plasmid pEXG2 (67) as described by Klein et al. (68). The primers used in this study are listed in Table S3.

### Generation of complementation constructs.

For complementation of the *ctpA*, *mepM1*, *mltG*, and *ygfB* mutant strains, the coding sequences were amplified by PCR from genomic DNA of ID40 and were assembled with the plasmid pJM220 (pUC18T-miniTn7T-gm-rhaSR-PrhaBAD) (69) by Gibson cloning. The constructed plasmids were transformed into E. coli SM10 λ *pir* and mobilized by conjugation into the mutant strains as described previously (70), with some modifications. A triparental mating was conducted by combining the recipient strain together with the mini-Tn*7*T-harboring SM10 λ *pir* strain and SM10 λ *pir*/pTNS3 harboring a Tn*7* transposase. Insertion of the mini-Tn*7*T construct into the *att*Tn*7* site was monitored by PCR. Excision of the pJM220 backbone containing the Gm resistance cassette was performed by expressing Flp recombinase from a conjugative plasmid, pFLP2. Finally, sucrose-resistant but Gm- and carbenicillin-sensitive colonies were verified by PCR.

### RNA isolation and qRT-PCR.

RNA isolation and quantitative reverse transcription-PCR (qRT-PCR) were performed as previously described (68).

### β-Lactamase activity assay.

A β-lactamase colorimetric activity assay (BioVision) based on nitrocefin turnover was performed according to the manufacturers’ instructions after dissolving the bacteria in 5-μl/mg β-lactamase assay buffer and diluting the supernatant of the sonified bacteria 1:50 in β-lactamase assay buffer.

### Antibiotic susceptibility testing.

For antibiotic susceptibility testing by broth microdilution, bacterial strains were grown overnight at 37°C in LB medium with or without 0.1% rhamnose. Physiological NaCl solution was inoculated to a McFarland standard of 0.5, and subsequently, 62.5 μl of the suspension was transferred into 15 ml Mueller-Hinton broth (with 0.1% rhamnose for the complemented strains) and mixed well. According to the manufacturer’s instructions, 50 to 100 μl of the suspension was transferred into each well of a broth microdilution microtiter plate (a Micronaut-S MHK Pseudomonas-2 [catalog number E1-099-100] or a Micronaut-S β-Lactamases [catalog number E1-111-040] plate [Merlin Diagnostika] or a Sensititre GN2F or Sensititre EUX2NF plate [Thermo Fisher Scientific]). The microtiter plates were incubated for 18 h at 37°C, and the OD_600_ was measured using a Tecan Infinite 200 Pro plate reader. Bacterial strains were considered sensitive to the respective antibiotic concentration if an OD_600_ value below 0.05 was measured.

Etests (Liofilchem) were conducted as previously described (68).

### Statistics.

Statistics were performed using GraphPad Prism (version 7.04) software, as described for each experiment in the table or figure legends.

### Data availability.

The whole genome and the plasmid sequences were annotated and submitted to the European Nucleotide Archive (ENA) (accession number PRJEB32702). Similarly, all TraDIS sequence data were uploaded to ENA (accession number PRJEB32702). A more detailed description of the files is provided in Table S4.

## Supplementary Material

Supplemental file 1

Supplemental file 2

Supplemental file 3

Supplemental file 4

## References

[1] Tacconelli E, Carrara E, Savoldi A, Harbarth S, Mendelson M, Monnet DL, Pulcini C, Kahlmeter G, Kluytmans J, Carmeli Y, Ouellette M, Outterson K, Patel J, Cavaleri M, Cox EM, Houchens CR, Grayson ML, Hansen P, Singh N, Theuretzbacher U, Magrini N, WHO Pathogens Priority List Working Group 2018 Discovery, research, and development of new antibiotics: the WHO priority list of antibiotic-resistant bacteria and tuberculosis. Lancet Infect Dis 18:318–327. doi:10.1016/S1473-3099(17)30753-3. 29276051

[2] Yoshimura F, Nikaido H 1982 Permeability of *Pseudomonas aeruginosa* outer membrane to hydrophilic solutes. J Bacteriol 152:636–642. 6813310 10.1128/jb.152.2.636-642.1982 PMC221510

[3] Strateva T, Yordanov D 2009 *Pseudomonas aeruginosa*—a phenomenon of bacterial resistance. J Med Microbiol 58:1133–1148. doi:10.1099/jmm.0.009142-0. 19528173

[4] Schmidtke AJ, Hanson ND 2008 Role of *ampD* homologs in overproduction of AmpC in clinical isolates of *Pseudomonas aeruginosa*. Antimicrob Agents Chemother 52:3922–3927. doi:10.1128/AAC.00341-08. 18779353 PMC2573135

[5] Tam VH, Schilling AN, LaRocco MT, Gentry LO, Lolans K, Quinn JP, Garey KW 2007 Prevalence of AmpC over-expression in bloodstream isolates of *Pseudomonas aeruginosa*. Clin Microbiol Infect 13:413–418. doi:10.1111/j.1469-0691.2006.01674.x. 17359326

[6] Moya B, Dötsch A, Juan C, Blazquez J, Zamorano L, Häussler S, Oliver A 2009 Beta-lactam resistance response triggered by inactivation of a nonessential penicillin-binding protein. PLoS Pathog 5:e1000353. doi:10.1371/journal.ppat.1000353. 19325877 PMC2654508

[7] Hanson ND, Sanders CC 1999 Regulation of inducible AmpC beta-lactamase expression among Enterobacteriaceae. Curr Pharm Des 5:881–894. 10539994

[8] Sanders CC, Sanders WE Jr. 1986 Type I beta-lactamases of gram-negative bacteria: interactions with beta-lactam antibiotics. J Infect Dis 154:792–800. doi:10.1093/infdis/154.5.792. 3490520

[9] Livermore DM 2002 The impact of carbapenemases on antimicrobial development and therapy. Curr Opin Investig Drugs 3:218–224. 12020049

[10] Papp-Wallace KM, Winkler ML, Taracila MA, Bonomo RA 2015 Variants of beta-lactamase KPC-2 that are resistant to inhibition by avibactam. Antimicrob Agents Chemother 59:3710–3717. doi:10.1128/AAC.04406-14. 25666153 PMC4468660

[11] Drawz SM, Bonomo RA 2010 Three decades of beta-lactamase inhibitors. Clin Microbiol Rev 23:160–201. doi:10.1128/CMR.00037-09. 20065329 PMC2806661

[12] Domalaon R, Brizuela M, Eisner B, Findlay B, Zhanel GG, Schweizer F 2019 Dilipid ultrashort cationic lipopeptides as adjuvants for chloramphenicol and other conventional antibiotics against Gram-negative bacteria. Amino Acids 51:383–393. doi:10.1007/s00726-018-2673-9. 30392097

[13] Lydon HL, Baccile N, Callaghan B, Marchant R, Mitchell CA, Banat IM 2017 Adjuvant antibiotic activity of acidic sophorolipids with potential for facilitating wound healing. Antimicrob Agents Chemother 61:e02547-16. doi:10.1128/aac.02547-16. 28242666 PMC5404594

[14] Maiden MM, Hunt AMA, Zachos MP, Gibson JA, Hurwitz ME, Mulks MH, Waters CM, Maiden MM, Hunt AMA, Zachos MP, Gibson JA, Hurwitz ME, Mulks MH, Waters CM 2018 Triclosan is an aminoglycoside adjuvant for eradication of *Pseudomonas aeruginosa* biofilms. Antimicrob Agents Chemother 62:e00146-18. doi:10.1128/aac.00146-18. 29661867 PMC5971565

[15] Baker KR, Jana B, Hansen AM, Vissing KJ, Nielsen HM, Franzyk H, Guardabassi L 2019 Repurposing azithromycin and rifampicin against Gram-negative pathogens by combination with peptide potentiators. Int J Antimicrob Agents 53:868–872. doi:10.1016/j.ijantimicag.2018.10.025. 30447380

[16] Dik DA, Madukoma CS, Tomoshige S, Kim C, Lastochkin E, Boggess WC, Fisher JF, Shrout JD, Mobashery S 2019 Slt, MltD, and MltG of *Pseudomonas aeruginosa* as targets of bulgecin A in potentiation of β-lactam antibiotics. ACS Chem Biol 14:296–303. doi:10.1021/acschembio.8b01025. 30620575 PMC8808744

[17] Jana B, Cain AK, Doerrler WT, Boinett CJ, Fookes MC, Parkhill J, Guardabassi L 2017 The secondary resistome of multidrug-resistant *Klebsiella pneumoniae*. Sci Rep 7:42483. doi:10.1038/srep42483. 28198411 PMC5309761

[18] Goodall ECA, Robinson A, Johnston IG, Jabbari S, Turner KA, Cunningham AF, Lund PA, Cole JA, Henderson IR, Goodall ECA, Robinson A, Johnston IG, Jabbari S, Turner KA, Cunningham AF, Lund PA, Cole JA, Henderson IR 2018 The essential genome of *Escherichia coli* K-12. mBio 9:e02096-17. doi:10.1128/mBio.02096-17. 29463657 PMC5821084

[19] Phan MD, Peters KM, Sarkar S, Lukowski SW, Allsopp LP, Gomes Moriel D, Achard ME, Totsika M, Marshall VM, Upton M, Beatson SA, Schembri MA 2013 The serum resistome of a globally disseminated multidrug resistant uropathogenic *Escherichia coli* clone. PLoS Genet 9:e1003834. doi:10.1371/journal.pgen.1003834. 24098145 PMC3789825

[20] Grant AJ, Oshota O, Chaudhuri RR, Mayho M, Peters SE, Clare S, Maskell DJ, Mastroeni P 2016 Genes required for the fitness of *Salmonella enterica* serovar Typhimurium during infection of immunodeficient *gp91*^−/−^ *phox* mice. Infect Immun 84:989–997. doi:10.1128/IAI.01423-15. 26787719 PMC4807482

[21] Hassan KA, Cain AK, Huang T, Liu Q, Elbourne LDH, Boinett CJ, Brzoska AJ, Li L, Ostrowski M, Nhu NTK, Nhu TDH, Baker S, Parkhill J, Paulsen IT, Hassan KA, Cain AK, Huang T, Liu Q, Elbourne LDH, Boinett CJ, Brzoska AJ, Li L, Ostrowski M, Nhu NTK, Nhu TDH, Baker S, Parkhill J, Paulsen IT 2016 Fluorescence-based flow sorting in parallel with transposon insertion site sequencing identifies multidrug efflux systems in *Acinetobacter baumannii*. mBio 7:e01200-16. doi:10.1128/mBio.01200-16. 27601573 PMC5013296

[22] Willmann M, Goettig S, Bezdan D, Macek B, Velic A, Marschal M, Vogel W, Flesch I, Markert U, Schmidt A, Kübler P, Haug M, Javed M, Jentzsch B, Oberhettinger P, Schütz M, Bohn E, Sonnabend M, Klein K, Autenrieth I, Ossowski S, Schwarz S, Peter S 2018 Multi-omics approach identifies novel pathogen-derived prognostic biomarkers in patients with *Pseudomonas aeruginosa* bloodstream infection. bioRxiv http://www.biorxiv.org/content/10.1101/309898v1.

[23] Larsen MV, Cosentino S, Rasmussen S, Friis C, Hasman H, Marvig RL, Jelsbak L, Sicheritz-Ponten T, Ussery DW, Aarestrup FM, Lund O 2012 Multilocus sequence typing of total-genome-sequenced bacteria. J Clin Microbiol 50:1355–1361. doi:10.1128/JCM.06094-11. 22238442 PMC3318499

[24] Zankari E, Hasman H, Cosentino S, Vestergaard M, Rasmussen S, Lund O, Aarestrup FM, Larsen MV 2012 Identification of acquired antimicrobial resistance genes. J Antimicrob Chemother 67:2640–2644. doi:10.1093/jac/dks261. 22782487 PMC3468078

[25] Ocampo-Sosa AA, Cabot G, Rodriguez C, Roman E, Tubau F, Macia MD, Moya B, Zamorano L, Suarez C, Pena C, Dominguez MA, Moncalian G, Oliver A, Martinez-Martinez L, Spanish Network for Research in Infectious Diseases (REIPI) 2012 Alterations of OprD in carbapenem-intermediate and -susceptible strains of *Pseudomonas aeruginosa* isolated from patients with bacteremia in a Spanish multicenter study. Antimicrob Agents Chemother 56:1703–1713. doi:10.1128/AAC.05451-11. 22290967 PMC3318371

[26] Shu JC, Kuo AJ, Su LH, Liu TP, Lee MH, Su IN, Wu TL 2017 Development of carbapenem resistance in *Pseudomonas aeruginosa* is associated with OprD polymorphisms, particularly the amino acid substitution at codon 170. J Antimicrob Chemother 72:2489–2495. doi:10.1093/jac/dkx158. 28535274

[27] Kim CH, Kang HY, Kim BR, Jeon H, Lee YC, Lee SH, Lee JC 2016 Mutational inactivation of OprD in carbapenem-resistant *Pseudomonas aeruginosa* isolates from Korean hospitals. J Microbiol 54:44–49. doi:10.1007/s12275-016-5562-5. 26727901

[28] El Amin N, Giske CG, Jalal S, Keijser B, Kronvall G, Wretlind B 2005 Carbapenem resistance mechanisms in *Pseudomonas aeruginosa*: alterations of porin OprD and efflux proteins do not fully explain resistance patterns observed in clinical isolates. APMIS 113:187–196. doi:10.1111/j.1600-0463.2005.apm1130306.x. 15799762

[29] Lee SA, Gallagher LA, Thongdee M, Staudinger BJ, Lippman S, Singh PK, Manoil C 2015 General and condition-specific essential functions of *Pseudomonas aeruginosa*. Proc Natl Acad Sci U S A 112:5189–5194. doi:10.1073/pnas.1422186112. 25848053 PMC4413342

[30] Fernández-Piñar R, Lo Sciuto A, Rossi A, Ranucci S, Bragonzi A, Imperi F 2015 In vitro and in vivo screening for novel essential cell-envelope proteins in *Pseudomonas aeruginosa*. Sci Rep 5:17593. doi:10.1038/srep17593. 26621210 PMC4665194

[31] Vötsch W, Templin MF 2000 Characterization of a beta-N-acetylglucosaminidase of *Escherichia coli* and elucidation of its role in muropeptide recycling and beta-lactamase induction. J Biol Chem 275:39032–39038. doi:10.1074/jbc.M004797200. 10978324

[32] Stubbs KA, Scaffidi A, Debowski AW, Mark BL, Stick RV, Vocadlo DJ 2008 Synthesis and use of mechanism-based protein-profiling probes for retaining beta-d-glucosaminidases facilitate identification of *Pseudomonas aeruginosa* NagZ. J Am Chem Soc 130:327–335. doi:10.1021/ja0763605. 18067297

[33] Acebron I, Mahasenan KV, De Benedetti S, Lee M, Artola-Recolons C, Hesek D, Wang H, Hermoso JA, Mobashery S 2017 Catalytic cycle of the N-acetylglucosaminidase NagZ from *Pseudomonas aeruginosa*. J Am Chem Soc 139:6795–6798. doi:10.1021/jacs.7b01626. 28482153 PMC6873925

[34] Cheng Q, Park JT 2002 Substrate specificity of the AmpG permease required for recycling of cell wall anhydro-muropeptides. J Bacteriol 184:6434–6436. doi:10.1128/jb.184.23.6434-6436.2002. 12426329 PMC135433

[35] Zamorano L, Reeve TM, Juan C, Moya B, Cabot G, Vocadlo DJ, Mark BL, Oliver A 2011 AmpG inactivation restores susceptibility of pan-beta-lactam-resistant *Pseudomonas aeruginosa* clinical strains. Antimicrob Agents Chemother 55:1990–1996. doi:10.1128/AAC.01688-10. 21357303 PMC3088256

[36] Zhang Y, Bao Q, Gagnon LA, Huletsky A, Oliver A, Jin S, Langaee T 2010 *ampG* gene of *Pseudomonas aeruginosa* and its role in beta-lactamase expression. Antimicrob Agents Chemother 54:4772–4779. doi:10.1128/AAC.00009-10. 20713660 PMC2976151

[37] Dötsch A, Becker T, Pommerenke C, Magnowska Z, Jansch L, Haussler S 2009 Genomewide identification of genetic determinants of antimicrobial drug resistance in *Pseudomonas aeruginosa*. Antimicrob Agents Chemother 53:2522–2531. doi:10.1128/AAC.00035-09. 19332674 PMC2687185

[38] Gisin J, Schneider A, Nagele B, Borisova M, Mayer C 2013 A cell wall recycling shortcut that bypasses peptidoglycan de novo biosynthesis. Nat Chem Biol 9:491–493. doi:10.1038/nchembio.1289. 23831760

[39] Borisova M, Gisin J, Mayer C 2014 Blocking peptidoglycan recycling in *Pseudomonas aeruginos*a attenuates intrinsic resistance to fosfomycin. Microb Drug Resist 20:231–237. doi:10.1089/mdr.2014.0036. 24819062 PMC4050453

[40] Borisova M, Gisin J, Mayer C 2017 The *N*-acetylmuramic acid 6-phosphate phosphatase MupP completes the *Pseudomonas* peptidoglycan recycling pathway leading to intrinsic fosfomycin resistance. mBio 8:e00092-17. doi:10.1128/mBio.00092-17. 28351914 PMC5371407

[41] Fumeaux C, Bernhardt TG 2017 Identification of MupP as a new peptidoglycan recycling factor and antibiotic resistance determinant in *Pseudomonas aeruginosa*. mBio 8:e00102-17. doi:10.1128/mBio.00102-17. 28351916 PMC5371409

[42] Cavallari JF, Lamers RP, Scheurwater EM, Matos AL, Burrows LL 2013 Changes to its peptidoglycan-remodeling enzyme repertoire modulate beta-lactam resistance in *Pseudomonas aeruginosa*. Antimicrob Agents Chemother 57:3078–3084. doi:10.1128/AAC.00268-13. 23612194 PMC3697359

[43] Srivastava D, Seo J, Rimal B, Kim SJ, Zhen S, Darwin AJ 2018 A proteolytic complex targets multiple cell wall hydrolases in *Pseudomonas aeruginosa*. mBio 9:e00972-18. doi:10.1128/mBio.00972-18. 30018106 PMC6050968

[44] Zincke D, Balasubramanian D, Silver LL, Mathee K 2016 Characterization of a carbapenem-hydrolyzing enzyme, PoxB, in *Pseudomonas aeruginosa* PAO1. Antimicrob Agents Chemother 60:936–945. doi:10.1128/AAC.01807-15. 26621621 PMC4750667

[45] Dhar S, Kumari H, Balasubramanian D, Mathee K 2018 Cell-wall recycling and synthesis in *Escherichia coli* and *Pseudomonas aeruginosa*—their role in the development of resistance. J Med Microbiol 67:1–21. doi:10.1099/jmm.0.000636. 29185941

[46] Mayer C 2019 Peptidoglycan recycling, a promising target for antibiotic adjuvants in antipseudomonal therapy. J Infect Dis 220:1713–1715. doi:10.1093/infdis/jiz378. 31325362

[47] Lamers RP, Nguyen UT, Nguyen Y, Buensuceso RN, Burrows LL 2015 Loss of membrane-bound lytic transglycosylases increases outer membrane permeability and beta-lactam sensitivity in *Pseudomonas aeruginosa*. Microbiologyopen 4:879–895. doi:10.1002/mbo3.286. 26374494 PMC4694138

[48] Yunck R, Cho H, Bernhardt TG 2016 Identification of MltG as a potential terminase for peptidoglycan polymerization in bacteria. Mol Microbiol 99:700–718. doi:10.1111/mmi.13258. 26507882 PMC4752859

[49] Sanz-García F, Hernando-Amado S, Martinez JL 2018 Mutation-driven evolution of *Pseudomonas aeruginosa* in the presence of either ceftazidime or ceftazidime-avibactam. Antimicrob Agents Chemother 62:e01379-18. doi:10.1128/AAC.01379-18. 30082283 PMC6153820

[50] Perley-Robertson GE, Yadav AK, Winogrodzki JL, Stubbs KA, Mark BL, Vocadlo DJ 2016 A fluorescent transport assay enables studying AmpG permeases involved in peptidoglycan recycling and antibiotic resistance. ACS Chem Biol 11:2626–2635. doi:10.1021/acschembio.6b00552. 27442597

[51] Jacobs C, Joris B, Jamin M, Klarsov K, Van Beeumen J, Mengin-Lecreulx D, van Heijenoort J, Park JT, Normark S, Frère JM 1995 AmpD, essential for both beta-lactamase regulation and cell wall recycling, is a novel cytosolic N-acetylmuramyl-l-alanine amidase. Mol Microbiol 15:553–559. doi:10.1111/j.1365-2958.1995.tb02268.x. 7783625

[52] Juan C, Macia MD, Gutierrez O, Vidal C, Perez JL, Oliver A 2005 Molecular mechanisms of beta-lactam resistance mediated by AmpC hyperproduction in *Pseudomonas aeruginosa* clinical strains. Antimicrob Agents Chemother 49:4733–4738. doi:10.1128/AAC.49.11.4733-4738.2005. 16251318 PMC1280133

[53] Langaee TY, Gagnon L, Huletsky A 2000 Inactivation of the ampD gene in *Pseudomonas aeruginosa* leads to moderate-basal-level and hyperinducible AmpC beta-lactamase expression. Antimicrob Agents Chemother 44:583–589. doi:10.1128/aac.44.3.583-589.2000. 10681322 PMC89730

[54] Feinbaum RL, Urbach JM, Liberati NT, Djonovic S, Adonizio A, Carvunis A-R, Ausubel FM 2012 Genome-wide identification of *Pseudomonas aeruginosa* virulence-related genes using a *Caenorhabditis elegans* infection model. PLoS Pathog 8:e1002813. doi:10.1371/journal.ppat.1002813. 22911607 PMC3406104

[55] Skurnik D, Roux D, Aschard H, Cattoir V, Yoder-Himes D, Lory S, Pier GB 2013 A comprehensive analysis of in vitro and in vivo genetic fitness of *Pseudomonas aeruginosa* using high-throughput sequencing of transposon libraries. PLoS Pathog 9:e1003582. doi:10.1371/journal.ppat.1003582. 24039572 PMC3764216

[56] Cabot G, Florit-Mendoza L, Sanchez-Diener I, Zamorano L, Oliver A 2018 Deciphering beta-lactamase-independent beta-lactam resistance evolution trajectories in *Pseudomonas aeruginosa*. J Antimicrob Chemother 73:3322–3331. doi:10.1093/jac/dky364. 30189050

[57] Peter S, Bosio M, Gross C, Bezdan D, Gutierrez J, Oberhettinger P, Liese J, Vogel W, Dörfel D, Berger L, Marschal M, Willmann M, Gut I, Gut M, Autenrieth I, Ossowski S 2019 Tracking of antibiotic resistance transfer and rapid plasmid evolution in a hospital setting by Nanopore sequencing. bioRxiv doi:10.1101/639609. PMC7440845 32817379

[58] Wick RR, Judd LM, Gorrie CL, Holt KE 2017 Unicycler: resolving bacterial genome assemblies from short and long sequencing reads. PLoS Comput Biol 13:e1005595. doi:10.1371/journal.pcbi.1005595. 28594827 PMC5481147

[59] Bankevich A, Nurk S, Antipov D, Gurevich AA, Dvorkin M, Kulikov AS, Lesin VM, Nikolenko SI, Pham S, Prjibelski AD, Pyshkin AV, Sirotkin AV, Vyahhi N, Tesler G, Alekseyev MA, Pevzner PA 2012 SPAdes: a new genome assembly algorithm and its applications to single-cell sequencing. J Comput Biol 19:455–477. doi:10.1089/cmb.2012.0021. 22506599 PMC3342519

[60] Seemann T 2014 Prokka: rapid prokaryotic genome annotation. Bioinformatics 30:2068–2069. doi:10.1093/bioinformatics/btu153. 24642063

[61] Kulasekara HD 2014 Transposon mutagenesis. Methods Mol Biol 1149:501–519. doi:10.1007/978-1-4939-0473-0_39. 24818929

[62] Barquist L, Mayho M, Cummins C, Cain AK, Boinett CJ, Page AJ, Langridge GC, Quail MA, Keane JA, Parkhill J 2016 The TraDIS toolkit: sequencing and analysis for dense transposon mutant libraries. Bioinformatics 32:1109–1111. doi:10.1093/bioinformatics/btw022. 26794317 PMC4896371

[63] Uren AG, Mikkers H, Kool J, van der Weyden L, Lund AH, Wilson CH, Rance R, Jonkers J, van Lohuizen M, Berns A, Adams DJ 2009 A high-throughput splinkerette-PCR method for the isolation and sequencing of retroviral insertion sites. Nat Protoc 4:789–798. doi:10.1038/nprot.2009.64. 19528954 PMC3627465

[64] Devon RS, Porteous DJ, Brookes AJ 1995 Splinkerettes—improved vectorettes for greater efficiency in PCR walking. Nucleic Acids Res 23:1644–1645. doi:10.1093/nar/23.9.1644. 7784225 PMC306912

[65] Dembek M, Barquist L, Boinett CJ, Cain AK, Mayho M, Lawley TD, Fairweather NF, Fagan RP 2015 High-throughput analysis of gene essentiality and sporulation in *Clostridium difficile*. mBio 6:e02383-14. doi:10.1128/mBio.02383-14. 25714712 PMC4358009

[66] Love MI, Huber W, Anders S 2014 Moderated estimation of fold change and dispersion for RNA-seq data with DESeq2. Genome Biol 15:550. doi:10.1186/s13059-014-0550-8. 25516281 PMC4302049

[67] Rietsch A, Vallet-Gely I, Dove SL, Mekalanos JJ 2005 ExsE, a secreted regulator of type III secretion genes in *Pseudomonas aeruginosa*. Proc Natl Acad Sci U S A 102:8006–8011. doi:10.1073/pnas.0503005102. 15911752 PMC1142391

[68] Klein K, Sonnabend MS, Frank L, Leibiger K, Franz-Wachtel M, Macek B, Trunk T, Leo JC, Autenrieth IB, Schutz M, Bohn E 2019 Deprivation of the periplasmic chaperone SurA reduces virulence and restores antibiotic susceptibility of multidrug-resistant *Pseudomonas aeruginosa*. Front Microbiol 10:100. doi:10.3389/fmicb.2019.00100. 30846971 PMC6394205

[69] Meisner J, Goldberg JB 2016 The *Escherichia coli* rhaSR-PrhaBAD inducible promoter system allows tightly controlled gene expression over a wide range in *Pseudomonas aeruginosa*. Appl Environ Microbiol 82:6715–6727. doi:10.1128/AEM.02041-16. 27613678 PMC5086565

[70] Choi KH, Schweizer HP 2006 Mini-Tn7 insertion in bacteria with single attTn7 sites: example *Pseudomonas aeruginosa*. Nat Protoc 1:153–161. doi:10.1038/nprot.2006.24. 17406227

