# Supplementary material for: Identification of Drug Resistance Determinants in a Clinical Isolate of Pseudomonas aeruginosa by High-Density Transposon Mutagenesis
Source: Antimicrob Agents Chemother. 2020 Feb 21;64(3):e01771-19. doi: 10.1128/AAC.01771-19 (PMC7038268; doi:10.1128/AAC.01771-19)
Supplement: Supplemental file 1 [file AAC.01771-19-s0001.pdf]

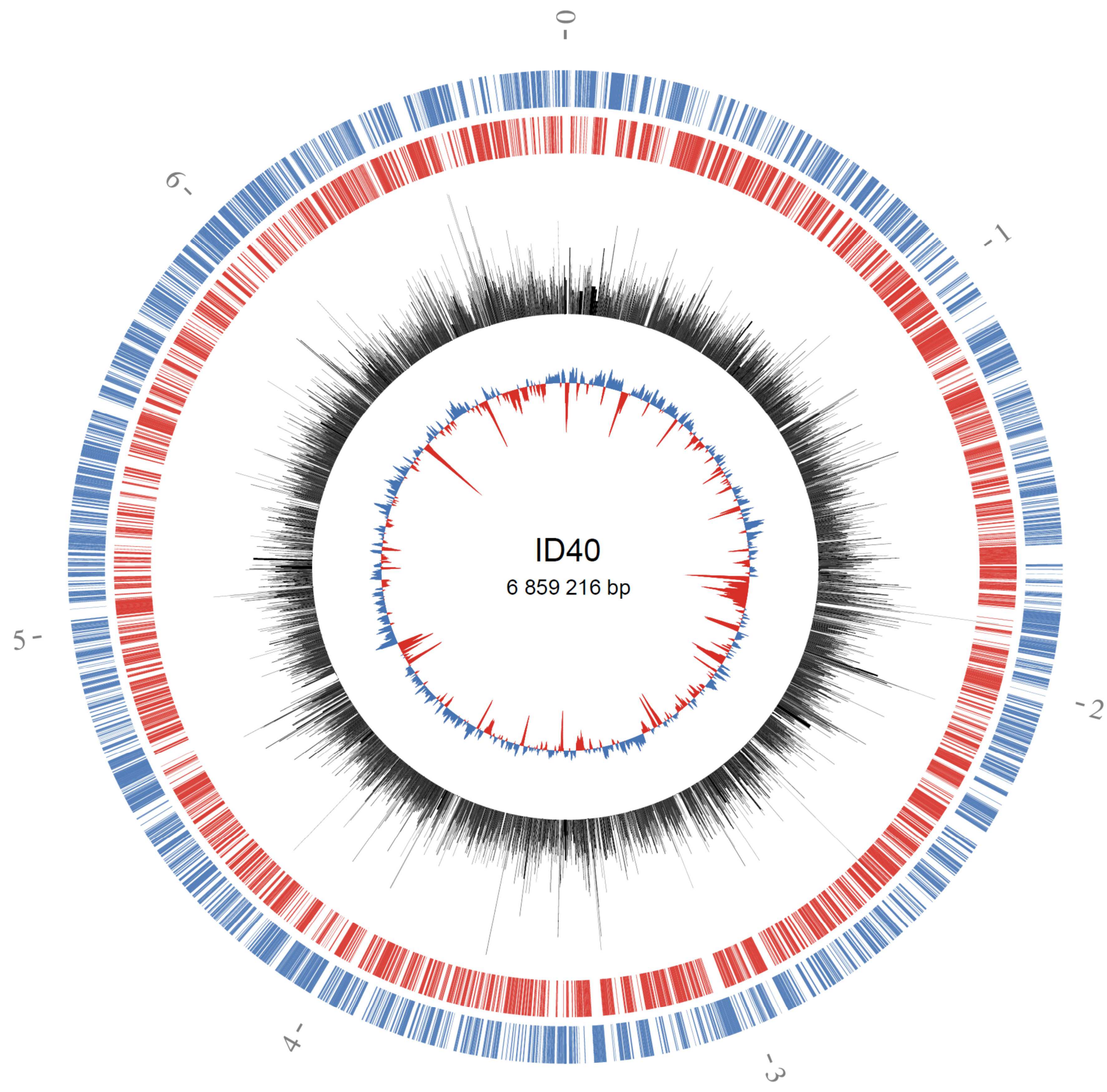

Figure S1. ID40 chromosome and coverage of Tn insertions.

The ID40 chromosome was visualized with BioCircos (1). Annotated genes are shown on the outer ring in red (antisense strand) and blue (sense strand). Tn insertions per gene were calculated for the combined LB samples and are shown on the middle ring (black). The height of the black bars corresponds to the number of Tn insertions per gene indicating a homogenous and complete coverage of the ID40 genome (except the genes essential under these conditions). Deviation from the average GC content is depicted on the inner ring (blue: higher GC content, red: lower GC content).

Table S1. Susceptibility of ID40 WT, deletion mutants and complementary strains against β-lactam antibiotics.

Minimal inhibitory concentrations (MICs) of ID40 WT and deletion mutants and complemented strains were determined by microbroth dilution or by E-Test for fosfomycin. The complemented strains (Δ*mltG*+, Δ*mepM*1+, Δ*ctpA*+, Δ*ygfB*+ and Δ*amgK*+) were induced with 0.1 % rhamnose. MIC values of the deletion mutants lower than that of the WT are highlighted in green and below the MIC breakpoint in bold green and light green background. MIC values higher compared to that of ID40 WT are highlighted in red.

	MIC Breakpoint (mg/L)		ID40 WT	Δ*mltG*	Δ*mltG*+	Δ*mepM1*	Δ*mepM1*+	Δ*ctpA*	Δ*ctpA*+	Δ*mepM1* Δ*ctpA*	Δ*ygfB*	Δ*ygfB*+	Δ*amgK*	Δ*amgK*+	Δ*tuaC*	PA14	PAO1	PAO1 Δ*amgK*
	S ≤	R >																
**MEM**	2	8	8	4	16	8	8	16	8	16	4	8	8	8	8	<0.5	<0.5	<0.5
**IMP**	4	4	32	**4**	32	32	16	32	32	32	8	32	8	16	32	<1	<1	<1
**FEP**	8	8	16	**4**	32	**4**	**8**	32	16	32	**8**	16	**8**	16	16	<1	<1	<1
**CAZ**	8	8	32	**2**	32	16	16	32	32	32	16	32	**8**	32	32	<1	<1	<1
**PIP**	16	16	128	**<4**	128	64	64	>128	128	128	32	128	32	64	128	<4	<4	<4
**TZP**	16	16	128	**4**	128	32	128	128	128	128	32	64	32	64	64	4	4	4
**ATM**	16	16	32	**2**	32	**16**	**16**	>32	32	32	**16**	32	**8**	**16**	>32	8	4	2
**FOS ***	-	-	96	96	64	96	96	96	64	64	128	64	48	96	96	48	128	32
**LEV**	1	1	>8	>8	>8	>8	>8	8	>8	>8	>8	>8	>8	>8	>8	0.25	0.5	0.5
**CIP**	0.5	0.5	8	4	4	8	4	4	4	8	8	8	4	4	4	0.125	2	**0.125**
**AMK**	8	16	8	8	8	8	8	8	8	8	8	8	8	8	8	<2	<2	<2
**TOB**	4	4	1	1	1	<0.5	<0.5	1	<0.5	1	1	1	<0.5	<0.5	1	<0.5	<0.5	<0.5
**GEN**	4	4	4	4	4	4	4	4	4	4	4	4	4	4	4	<1	<1	<1
**COL**	4	4	4	4	2	4	2	2	2	2	2	4	2	2	2	1	2	2

MEM, meropenem; IMP, imipenem; FEP, cefepime; CAZ, ceftazidime; PIP, piperacillin; TZP, piperacillin/tazobactam; ATM, aztreonam; FOS, fosfomycin; LEV, levofloxacin; CIP, ciprofloxacin; AMK, amikacin; TOB, tobramycin; GEN, gentamicin; COL, colistin;

*E-Test

Table S2. Strains and plasmids

Strain	Relevant characteristics	Source
Pseudomonas aeruginosa		
ID40	Clinical isolate from bloodstream infection, antibiotic resistance comprises resistance against, cefepime, ceftazidim, imipenem, ciprofloxacin, levofloxacin, piperacillin and piperacillin/tazobactam	(2)
ygfB (TUEID40_03245)	In-frame deletion mutant of ID40 encoding the first 10 and last 10 amino acids of the specific CDS	This study
ctpA (TUEID40_03143)		
mepM1 (TUEID40_04290)		
mltG (TUEID40_05736)		
amgK (TUEID40_04233)		
tuaC (TUEID40_05543)		
ctpA mepM1	Double in-frame deletion mutant of ID40 encoding the first 10 and last 10 amino acids of *ctpA* and *mepM1* CDS	
ygfB YgfB+	Mutant strain complemented with plasmid pJM220 harbouring the specific CDS; Gm^R	
ctpA CtpA+		
mepM1 MepM1+		
mltG MltG+		
amgK AmgK+		
Escherichia coli		
SM10 λ pir	thi thr leu tonA lacY supE recA::RP4-2-Tc::Mu Km λpir	(3)
**Plasmids**		
pEXG2	Allelic exchange vector with pBR origin, Gm^R, $sacB^+$	(4)
pEXG2Δ*ygfB* mutator	pEXG2 derivative for in-frame deletion of the specific CDS ; Gm^R	This study
pEXG2Δ*ctpA* mutator		
pEXG2Δ*mepM1* mutator		
pEXG2Δ*mltG* mutator		
pEXG2Δ*tuaC* mutator		
pEXG2 Δ*amgK* mutator		
pJM220 (pUC18T-miniTn7T-gm-rhaSR-PrhaBAD)	mini-Tn7 base vector with transcriptional terminators, rhamnose inducible promoter and MCS; Gm^R	(5)
pJM220*ygfB*	pJM220 derivative for complementation of the specific CDS ; Gm^R	This study
pJM220*ctpA*		
pJM220*mepM1*		
pJM220*mltG*		
pJM220*amgK*		
pTNS3	Amp^R; plasmid expressing tnsABCD from P1 and Plac	(6)
pFLP2	Cb^R/Amp^R; $sacB^+$; Flp recombinase	(7)
pBT20	Gm^R, Himar1 Mariner C9, transposon cassette	(8)

Amp, Ampicillin; Cb, Carbenicillin; Gm, Gentamicin; Km, Kanamycin

Table S3. Oligonucleotides

Name	Sequence 5’-3’
**Primer for Gibson Cloning**	
gib_uni_pEXG2_f	AGGTCGACTCTAGAGGATCC
gib_uni_pEXG2_r	TTCCGGCTCGTATAATGTGT
pEXG2_seq_f	TACTGTGTTAGCGGTCTG
pEXG2_seq_r	GATCCGGAACATAATGGTG
pEXG2_ygfB_up_f	AGCTAATTCCACACATTATACGAGCCGGAAGTAGCAGT CGATCTCGCAG
pEXG2_ ygfB _up_r	CTCGCCGAGGCGGCCATGCCGGTCTCGCCGCCTTCAC TGCACTGAGGTTTC
pEXG2_ ygfB _dn_f	GATCATGCAGAAACCTCAGTGCAGTGAAGGCGGCGAGA CCGGCATGGC
pEXG2_ ygfB _dn_r	TCGAGCCCGGGGATCCTCTAGAGTCGACCTATCACCGA CTGGCCAACACACC
ygfB_seq_f	CATGACCTTCACTTCGTTG
ygfB _seq_r	CTTGTCGAGAATCTGCAC
ygfB_inside_r	GGAAGTCATGGAATACCTG
pEXG2_ctpA_up_f	AGCTAATTCCACACATTATACGAGCCGGAAGGTATTCGC CGACTGGTTGC
pEXG2_ctpA_up_r	CGCGGAACGCCGACATCAGTTGCCGCGGGTGAAGCAA TGCAGCATGTTCAGC
pEXG2_ctpA_dn_f	TGATTAGGAGCTGAACATGCTGCATTGCTTCACCCGCG GCAACTGATGTC
pEXG2_ctpA_dn_r	TCGAGCCCGGGGATCCTCTAGAGTCGACCTGCTCTTCC TGGGTCAGCTC
ctpA_seq_f	CTACCTCAGCCTTTATGGC
ctpA_seq_r	ATCGGCATGTGCAGGATCA
ctpA_inside_r	GATCGGCGAGACCACCTTGA
pEXG2_mepM_up_f	AGCTAATTCCACACATTATACGAGCCGGAACAGCAGCG ATTCGCTGATG
pEXG2_mepM_up_r	AGTTTGGCACTACTGGTGTTCCCCTCGAGCAACAAGCA GCGCTGATCGTG
pEXG2_mepM_dn_f	GGTAGCGCGGCACGATCAGCGCTGCTTGTTGCTCGAG GGGAACACCAG
pEXG2_mepM_dn_r	TCGAGCCCGGGGATCCTCTAGAGTCGACCTATCCAGCC CTTCCAGCAAC
mepM_seq_f	GTTGGTGATGAATCCAGG
mepM_seq_r	GAGGATCTTGAAGACCTG
mepM_inside_r	ACGAGTTCCAGATCAATGG
pEXG2_mltG_up_f	AGCTAATTCCACACATTATACGAGCCGGAAAGCACGAA GTAGTCCGGATGG
pEXG2_mltG_up_r	GGATTTCTGACTTTGATGCGCAAACTGCTGCCGCCGCC ACAATGAGCG
pEXG2_mltG_dn_f	TCTTGGCTGCGCCGCTCATTGTGGCGGCGGCAGCAGTT TGCGCATCAAAGTC

pEXG2_mltG_dn_r	TCGAGCCCGGGGATCCTCTAGAGTCGACCTTCGAACTC GTTCGGTTTCGG
mltG_seq_f	GATATCAGCCATTCAGACG
mltG_seq_r	GCCAAGCTGATCGTCAC
mltG_inside_r	TGAGACCCTGTATTTCGTG
anmK_up_f	AAGCTAATTCCACACATTATACGAGCCGGAAGGTCGGT GACATGAACCTCAAGG
anmK_up_r	GTGCCGCGCTACCTGGGGTTGATGTCCGGAAGAACTCT GGGCGCGCTG
anmK_dn_f	TCAGGCGGGATACAGCGCGCCCAGAGTTCTTCCGGAC ATCAACCCCAGG
anmK_dn_r	CTCGAGCCCGGGGATCCTCTAGAGTCGACCTCCAGCTC GCTGTTCGTTG
anmK_seq_f	GGACCCGATGAGCTTCCAG
anmK_seq_r	TGATCGCGCGCATGGATC
anmK_inside_r	CAGTACCGACGAATACGGCATTC
amgK_up_f	AGCTAATTCCACACATTATACGAGCCGGAACACCAGCAT GCCCTTGTCG
amgK_up_r	GGGACGGCGCGCCACGGCGGTTTCCAGATACAGCTGC TGGAAACGGGC
amgK_dn_f	ATGTCTGATGATGCCCGTTTCCAGCAGCTGTATCTGGAA ACCGCCGTGGC
amgK_dn_r	TCGAGCCCGGGGATCCTCTAGAGTCGACCTAACCTGCG GTCCAGCAGTTG
amgK_seq_f	TGTTAATCCGGGCTTCCTGCG
amgK_seq_r	CCGGCGGCGAGGATCATC
amgK_inside_r	CCTGCCAGCGGAAATAACGAC
pEXG2_tuaC_up_f	AGCTAATTCCACACATTATACGAGCCGGAAACCCTTACC AAGCATTCTTGC
pEXG2_tuaC_up_r	TAAGGAAGGCCGGCGTTGAGCTATTTCAATAGGGGAGA CTCATGAGCAGAA
pEXG2_tuaC_dn_f	TCACCAGGATTCTGCTCATGAGTCTCCCCTATTGAAATA GCTCAACGCCGG
pEXG2_tuaC_dn_r	TCGAGCCCGGGGATCCTCTAGAGTCGACCTTACGGTTA TTCTCAACTGCCTC
tuaC _seq_f	GCTTCTTCGATTGTCAACC
tuaC _seq_r	GTATATGGTTCAGGAATGC
tuaC _inside_r	CAGCTATTAGGTGATGAGC
Gib_uni_pJM220_f	TACCTCGCGAAGGCCTTGCA
Gib_uni_pJM220_r	AAGCTTCTCGAGGAATTCCTGC
Tn7R_f	CACAGCATAACTGGACTGATTTC
glmS_down_f	TCCATGCCGAAGCCTACCC
pJM220_ygfB_f	CTAGTGCTCTGCAGGAATTCCTCGAGAAGCTTATGCCG GTCTCGCCGGCC
pJM220_ygfB_r	CTGGTTGGCCTGCAAGGCCTTCGCGAGGTATCAGTGCA GTGAAGGCTTGG

pJM220_ctpA_f	ATGAAATTCAACTAGTGCTCTGCAGGAATTCCTCGAGAAGCTTATGCTGCATTGCTTCCGTCC
pJM220_ctpA_r	TTTGGAACTAGATTTCACTTATCTGGTTGGCCTGCAAGGCCTTCGCGAGGTATCAGTTGCCGCGGGTGAC
pJM220_ctpA_seq_f	AAGAGCCAGGTCCTCGAACC
pJM220_mepM1_f	AGTGCTCTGCAGGAATTCCTCGAGAAGCTTGTGTTCCCCTCGAGCGAAGT
pJM220_mepM1_r	ATCTGGTTGGCCTGCAAGGCCTTCGCGAGGTATCAGCGCTGCTTGTTCAG
pJM220_mepM1_seq_f	AAGCTCGACCCCAAAGGAGA
pJM220_mltG_f	CTAGTGCTCTGCAGGAATTCCTCGAGAAGCTTATGCGCAAACTGCTGGTG
pJM220_mltG_r	ATCTGGTTGGCCTGCAAGGCCTTCGCGAGGTATCATTGTGGCGGCGGGGT
pJM220_mltG_seq_f	GGCGAGATCATGCAACGC
pJM220_anmK_f	CTAGTGCTCTGCAGGAATTCCTCGAGAAGCTTGTGCCGCGCTACCTGGGG
pJM220_anmK_r	CTGGTTGGCCTGCAAGGCCTTCGCGAGGTATCAGGCGGGATACAGCGC
pJM220_amgK_f	AGTGCTCTGCAGGAATTCCTCGAGAAGCTTATGTCTGATGATGCCCGTTTCCA
pJM220_amgK_r	CTTATTCAGGCGTAGCAC
Tn7R_f	CACAGCATAACTGGACTGATTTC
pFPL2_sacB_f	AGACCGCTAACACAGTACAT
pFPL2_sacB_r	TCGGCATTTTCTTTTGCGTT
**Primer for qRT-PCR**	
PA14_gyrB_f	CGTAACCTGAACAACTACATCGAG
PA14_gyrB_r	AAGTACTTGCCCATCTCCTGTTC
ID40_ampC_f	TGCTGCTCCATGAGTCGTTC
ID40_ampC_r	CGCCTCTATTCCAACCCGAG
ID40_OXA_f	TTCGCCCTGAACATCGACAT
ID40_OXA_r	GCAGTATCCCGAGAGCCTTG

Table S4. Files uploaded to ENA

ID40 chromosome and plasmid sequence

Study: PRJEB32702

Accession number:

Name	Accession number
ID40 chromosome	LR700248
ID40 plasmid	LR700249

TraDIS experiment sequences

Study: PRJEB32702

Experiment accession number:

Name	Run number
LB replicate 1	ERX3418260
FEP replicate 1	ERX3418257
MEM replicate 1	ERX3418263
LB replicate 2	ERX3418261
FEP replicate 2	ERX3418258
MEM replicate 2	ERX3418264
LB replicate 3	ERX3418262
FEP replicate 3	ERX3418259
MEM replicate 3	ERX3418265

References

1. **Cui Y, Chen X, Luo H, Fan Z, Luo J, He S, Yue H, Zhang P, Chen R.** 2016. BioCircos.js: an interactive Circos JavaScript library for biological data visualization on web applications. Bioinformatics 32:1740-2.doi:10.1093/bioinformatics/btw041. http://www.ncbi.nlm.nih.gov/pubmed/26819473.

2. **Willmann M, Goettig S, Bezdan D, Macek B, Velic A, Marschal M, Vogel W, Flesch I, Markert U, Schmidt A, Kübler P, Haug M, Javed M, Jentzsch B, Oberhettinger P, Schütz M, Bohn E, Sonnabend M, Klein K, Autenrieth I, Ossowski S, Schwarz S, Peter S.** 2018. Multi-omics approach identifies novel pathogen-derived prognostic biomarkers in patients with *Pseudomonas aeruginosa* bloodstream infection. biorxiv doi:10.1101/309898.doi:10.1101/309898. http://www.biorxiv.org/content/10.1101/309898v1.

3. **Simon R, Priefer U, Puhler A.** 1983. A Broad Host Range Mobilization System for Invivo Genetic-Engineering - Transposon Mutagenesis in Gram-Negative Bacteria. Bio-Technology 1:784-791.doi:Doi 10.1038/Nbt1183-784. <Go to ISI>://WOS:A1983RQ64200021.

4. **Rietsch A, Vallet-Gely I, Dove SL, Mekalanos JJ.** 2005. ExsE, a secreted regulator of type III secretion genes in *Pseudomonas aeruginosa*. Proceedings of the National Academy of Sciences of the United States of America 102:8006-11.doi:10.1073/pnas.0503005102. http://www.ncbi.nlm.nih.gov/pubmed/15911752.

5. **Meisner J, Goldberg JB.** 2016. The *Escherichia coli* rhaSR-PrhaBAD Inducible Promoter System Allows Tightly Controlled Gene Expression over a Wide Range in *Pseudomonas aeruginosa*. Applied and environmental microbiology 82:6715-6727.doi:10.1128/AEM.02041-16. http://www.ncbi.nlm.nih.gov/pubmed/27613678.

6. **Choi KH, Mima T, Casart Y, Rholl D, Kumar A, Beacham IR, Schweizer HP.** 2008. Genetic tools for select-agent-compliant manipulation of Burkholderia pseudomallei. Applied and environmental microbiology 74:1064-75.doi:10.1128/AEM.02430-07. http://www.ncbi.nlm.nih.gov/pubmed/18156318.

7. **Hoang TT, Kutchma AJ, Becher A, Schweizer HP.** 2000. Integration-proficient plasmids for *Pseudomonas aeruginosa:* site-specific integration and use for engineering of reporter and expression strains. Plasmid 43:59-72.doi:10.1006/plas.1999.1441. http://www.ncbi.nlm.nih.gov/pubmed/10610820.

8. **Kulasekara HD.** 2014. Transposon mutagenesis. Methods in molecular biology 1149:501-19.doi:10.1007/978-1-4939-0473-0_39. http://www.ncbi.nlm.nih.gov/pubmed/24818929.
